# Genome-Wide microRNA Profiling Using Oligonucleotide Microarray Reveals Regulatory Networks of microRNAs in *Nicotiana benthamiana* During Beet Necrotic Yellow Vein Virus Infection

**DOI:** 10.3390/v12030310

**Published:** 2020-03-12

**Authors:** Junying Liu, Huiyan Fan, Ying Wang, Chenggui Han, Xianbing Wang, Jialin Yu, Dawei Li, Yongliang Zhang

**Affiliations:** 1 State Key Laboratory for Agro-Biotechnology and Ministry of Agriculture Key Laboratory of Pest Monitoring and Green Management, College of Plant Protection, China Agricultural University, Beijing 100193, China; ljyqau@163.com (J.L.); huiyanfan@126.com (H.F.); yingwang@cau.edu.cn (Y.W.); 2 Laboratory of Phytopathology, College of Chemistry Biology and Environment, Yuxi Normal University, Yuxi 653100, China; 3 State Key Laboratory of Agro-Biotechnology and Ministry of Agriculture Key Laboratory of Soil Microbiology, College of Biological Sciences, China Agricultural University, Beijing 100193, China; wangxianbing@cau.edu.cn (X.W.); yjl@cau.edu.cn (J.Y.); dawei.li@cau.edu.cn (D.L.)

**Keywords:** *Beet necrotic yellow vein virus*, *Nicotiana benthamiana*, microarray, microRNAs, hormone signaling, superoxide free radicals O_2_^−^, reactive oxygen intermediates, defense

## Abstract

*Beet necrotic yellow vein virus* (BNYVV) infections induce stunting and leaf curling, as well as root and floral developmental defects and leaf senescence in *Nicotiana benthamiana*. A microarray analysis with probes capable of detecting 1596 candidate microRNAs (miRNAs) was conducted to investigate differentially expressed miRNAs and their targets upon BNYVV infection of *N. benthamiana* plants. Eight species-specific miRNAs of *N. benthamiana* were identified. Comprehensive characterization of the *N. benthamiana* microRNA profile in response to the BNYVV infection revealed that 129 miRNAs were altered, including four species-specific miRNAs. The targets of the differentially expressed miRNAs were predicted accordingly. The expressions of miR164, 160, and 393 were up-regulated by BNYVV infection, and those of their target genes, *NAC21/22*, *ARF17/18*, and *TIR*, were down-regulated. *GRF1*, which is a target of miR396, was also down-regulated. Further genetic analysis of *GRF1*, by *Tobacco rattle virus*-induced gene silencing, assay confirmed the involvement of *GRF1* in the symptom development during BNYVV infection. BNYVV infection also induced the up-regulation of miR168 and miR398. The miR398 was predicted to target *umecyanin*, and silencing of *umecyanin* could enhance plant resistance against viruses, suggesting the activation of primary defense response to BNYVV infection in *N. benthamiana*. These results provide a global profile of miRNA changes induced by BNYVV infection and enhance our understanding of the mechanisms underlying BNYVV pathogenesis.

## 1. Introduction

Beet necrotic yellow vein virus (BNYVV), a member of the *Benyvirus* genus, is a multipartite virus containing four or five single-stranded positive-sense RNAs that are individually packaged into rod-shaped virions [1,2,3]. Among RNA1–5, RNA1 and RNA2, which are essential for infection and virus replication, encode the housekeeping genes, such as *replicase,* coat protein, and triple gene block movement protein; the smaller RNAs, RNA3, -4, and -5, are involved in pathogenicity or vector transmission during systemic BNYVV infection of beet or *Nicotiana benthamiana* [4,5]. RNA3 encodes the p25 protein. Although, p25 is a pathogenicity determinant in sugar beet, it has no pronounced effect in *N. benthamiana* during BNYVV infection [6]. In BNYVV-infected *Beta macrocarpa*, some differentially expressed microRNAs (miRNAs), involved in auxin biosynthesis and signal transduction pathways, may function in restraining plant growth; up-regulate miR156 in the ‘miR156–squamosa promoter-binding-like protein–miR172–AP2-like factor’ pathway may be responsible for abnormal axillary bud development [7]. Transcriptome sequencing indicated that changes in the differently expressed genes are mainly enrichment in response to biotic stimulus and primary metabolic process [8]. RNA4 encodes a root-specific silencing suppressor p31 that is involved in efficient vector transmission and slight enhancement of symptom expression in some *Beta* species, but it is a pathogenicity determinant in *N. benthamiana* [6]. Our recent studies showed that RNA4-encoded p31 is responsible for the induction of *PR-10* transcription, which was closely associated with the induction of stunting and leaf curling symptoms [9]. 

We characterized the transcriptome of *N. benthamiana* and *B. macrocarpa* leaves in response to BNYVV infection, using the deep sequencing previously reported [8,10]. A study extended this research by conducting transcriptomic analysis of naturally infected sugar beet (*Beta vulgaris*) roots [11]. A comparison of these studies revealed that some signaling pathways, such as the pathogenesis-related defense, cell-wall-associated, and hormone biosynthesis, are regulated in all of these different hosts [8,10,11,12], suggesting evolutionarily conserved responses of plants to BNYVV infection. However, some genes showed diverse expression patterns in different tissues across the experimental and natural hosts. For example, expansin-related genes were up-regulated in the leaves of *B. macrocarpa* and the root of *B. vulgaris* [8,11,12], but were repressed in the BNYVV-infected *N. benthamiana* leaves [10], suggesting that the gene expression in response to BNYVV infection is tissue-dependent and also varies among distantly related plant species.

MicroRNAs are important and conserved regulators that function in various plant physiological and developmental processes. Plant miRNAs are a class of 20–24 nt endogenous small non-coding RNAs. Coding genes for miRNAs possess their own transcriptional units that are regulated by the corresponding transcriptional activator [13]. These miRNA genes (*MIR*) are transcribed into primary miRNAs with a secondary stem-loop structure in the partial sequence by RNA polymerase II, and then primary miRNAs are processed into miRNA-3p/miRNA-5p duplex by the Dicer-like protein complex [14,15]. After transport into the cytoplasm and being methylated, miRNA-3p or miRNA-5p enters into the RNA-induced silencing complex (RISC) and modulates plant morphogenesis and growth [16,17]. For example, miR156 is a switch between vegetative and reproductive growth in plants through acting as the miR156–squamosa promoter-binding-like protein–miR172–AP2-like factor [18,19]. miR164 is involved in the EIN2–EIN3–miR164–NAC2 signaling module to regulate leaf senescence [20].

Plant viruses selectively regulate the expression of several miRNAs to counteract the plant defenses, leading to the formation of virus-induced symptoms. For example, *Rice stripe virus* (RSV) blocks the defense response by significantly upregulating miR1870-5p and miR1423-5p during infection of rice plants [21]. *Rice ragged stunt virus* (RRSV) suppresses jasmonic acid (JA)-mediated defenses by inducing the expression of miR319 in rice, resulting in the enhancement of viral infection and symptom development [22]. Similarly, mis-regulation of miR167 and its target gene, *auxin response factor 8*, is responsible for the developmental abnormalities caused by three distinct viral silencing suppressors, HC-Pro, P19, and P15, in *Arabidopsis* [23]. 

As obligate intracellular parasites, plant viruses depend on cellular machinery to support their propagation. Plant viruses often perturb the host plants’ hormone signaling pathways to facilitate their infection and symptom induction. For example, *Rice* *dwarf* *virus* (RDV) P2 protein interacts with *ent-kaurene oxidases* to reduce the biosynthesis of gibberellins and promotes the appearance of the rice dwarf symptom. RDV hijacks auxin signaling by directly targeting the rice OsIAA10 protein, enhancing viral infection and disease development [24,25]. *Tobacco mosaic virus*-Cg coat protein stabilizes DELLA proteins which is named after its N-terminal containing DELLA domain, resulting in slow growth and flowering delays [26]. Similarly, *Tobacco mosaic virus* replication protein can interact with the Aux/IAA protein PAPI/IAA26 to regulate the disease development [27,28].

Although the symptoms in *N. benthamiana*, characterized by the developmental defects in root, leaves, and flowers during BNYVV infection, have been reported previously [6], whether miRNAs and hormones play roles in the development of symptoms remains largely unknown. In this study, based on microarray analysis and previously reported transcriptome sequencing [10], a systematic analysis of miRNA and hormone signaling pathways was conducted to explore their possible roles in BNYVV-induced symptoms.

## 2. Materials and Methods 

### 2.1. Plants, Viral Inoculations, and Detection

*N. benthamiana* plants were grown in a controlled-environment chamber at 24 ± 1 °C with 16 h of illumination and 8 h darkness per day. Hydroponics of *N. benthamiana* was conducted by using Hoagland’s nutrient solution, according to previous methods [29]. BN12 (RNAs 1 and 2), BN123 (RNAs 1, 2, and 3), BN124 (RNAs 1, 2, and 4), and BN1234 (RNAs 1, 2, 3, and 4) were preserved in our laboratory [30]. In vitro transcripts of BNYVV RNAs (1 μg/μL) were then mixed with equal volumes of inoculation buffer (50 mM glycine, 30 mM K_2_HPO_4_, 1% bentonite, and 1% celite, pH 9.2), and rubbed onto leaves of *N. benthamiana* (20 μL per leaf). Inoculation buffer without the addition of RNAs served as the negative control. Three pieces of leaves per plant were inoculated. At 12 days post inoculation (dpi), Western blot analyses of total protein extracts from the upper un-inoculated leaves was conducted using rabbit polyclonal antibodies against BNYVV coat protein (Figure S7).

### 2.2. Total RNA Extraction

The total RNAs were extracted from *N. benthamiana* leaves at 12 dpi according to previously described methods [10]. RNA integrity and size distribution were examined via a Bioanalyzer 2100 (Agilent Technologies, Palo Alto, CA, USA) and agarose gel electrophoresis (1%). For each group, the RNA pool was prepared by mixing RNA samples (12 μg per sample) from three individual plants.

### 2.3. Microarray Analysis

Based on the *N. benthamiana* small RNA library (GenBank accession No. GSE80694) [31] and the reported *N. benthamiana* genome database [32], miRNAs were predicted with the ACGT101-miR-v3.5 software package (LC Sciences, Houston, TX, USA), followed by analysis with previously described procedures [33]. We obtained 1596 candidate miRNAs and the same number of probes were designed and synthesized for the subsequent microarray analysis.

The miRNA microarray experiment was performed according to the protocol provided by LC Sciences (Hangzhou, China). Briefly, 1596 probes were designed for the miRNA microarray including 689 known miRNAs belonging to 86 miRNA families from 19 species, and 907 novel miRNAs from detailed prediction performed by LC Sciences (Table S1). 

The small RNAs (<300 nt) were size-fractionated using Microcon YM-100 centrifugal filters (Millipore, Bedford, MA) from 2 µg of total RNA samples. These small RNAs were extended at the 3′ end with a poly (A) tail by using poly (A) polymerase. An oligonucleotide tag was ligated to the poly (A) tail for later fluorescent dye staining. We made 1596 antisense detection probes using in situ synthesis using photo-generated reagent chemistry. Hybridization was performed overnight on a µParaflo microfluidic chip using a micro-circulation pump (Atactic Technologies, Houston, TX, USA). 

Each probe was used for three times, arranged in different places on one chip, and three biological replicates were performed. Tag-specific Cy3 and Cy5 dyes were used to label virus-free and BN1234-infected RNA samples, respectively, and were exchanged during the double-color microarray assays. After RNA hybridization, tag-conjugating Cy3 and Cy5 dyes were circulated through the microfluidic chip for dye staining. Fluorescence images were recorded using a laser scanner (GenePix 4000B, Molecular Device, Union City, CA, USA) and digitized using Array-Pro image analysis software (Media Cybernetics, Silver Spring, MD, USA). The background signals were subtracted from the original data, followed by normalization of the signals using the locally weighted regression (LOWESS) filter.

### 2.4. Target Prediction of Differentially Expressed miRNAs

The differentially expressed miRNA targets were annotated using the standard settings of psRNATarget [34] with a maximum expectation value of 3.0 using transcripts of *N. benthamiana* (Niben101) as references. GO analysis was performed to annotate the predicted targets.

### 2.5. Quantitative Reverse-Transcription Real-Time PCR (qRT-PCR)

To validate the small RNA sequencing results, the relative expression levels of 15 selected miRNAs from BN1234-infected or mock-inoculated leaves were validated using stem-loop qRT-PCR. Primers for the stem-loop qRT-PCR were designed using methods previously described [35]. Total RNA (3 μg) was used in the reverse transcription reaction (30 μL). qPCR was performed in 96-well plates using the CFX96 real-time PCR detection system (Bio-Rad, Hercules, CA, USA) with the following parameters: 95 °C for 15 s, followed by 40 cycles of 95 °C for 15 s, and then annealing at 60 °C for 30 s. Each reaction mixture consisted of 1 µL cDNA, 7 µL SoFast EyaGreen Supermix (Bio-Rad, Hercules, CA, USA), 1.5 µL (3 pmol/µL) of both forward and reverse primers, and 3 µL PCR-grade water [10].

The transcript levels of several target genes of selected six miRNAs were also assayed by qRT-PCR. Reverse transcription of RNA was conducted using oligo (dT)_20_. qPCR was conducted as described above. *EF1A* was used as an internal control. All reactions were performed using three biological replicates for each treatment, and each biological sample of three pooled plants was evaluated with three technical replicates. All primers, used in this study, are listed in Table S3.

### 2.6. Virus-Induced Gene Silencing (VIGS) of Selected Targets

TRV-based silencing of target genes was performed according to previously described methods [36]. The gene fragments were PCR amplified with specific primers (Table S3), digested with *Sma*I/*Kpn*I, and ligated into similarly digested pYL156 vector. TRV:mCherry, containing about 400 bp of DNA fragment from mCherry fluorescent protein, served as the control. To confirm the systemic infection of TRV, Western blot analysis was used for the extracts from systemic leaves of TRV:X-inoculated *N. benthamiana* using the antibodies against the TRV coat protein (Figure S8). At 6 dpi, the expression level of various target genes was analyzed by qRT-PCR with the primers listed in Table S3. *EF1A* was used as the internal reference gene. At 7 dpi, the systemic leaves of TRV:X-infected *N. benthamiana* were inoculated with BN124. At 25 dpi, Western blot was performed to detect the CP accumulation in the inoculated leaves.

### 2.7. Determination of ROS Levels

ROS levels of systemic leaves of TRV:X-treated plants were determined as described previously [37]. Superoxide free radicals were detected with 0.1 mg/mL nitroblue tetrazolium (Sigma) in 25 mM HEPES buffer (pH 7.6), and hydrogen peroxide (H_2_O_2_) was detected by 3,3′-diaminobenzidine staining.

## 3. Results

### 3.1. Floral and Root Development Defects During BNYVV Infection Requires the Presence of RNA4

**Figure 1:**
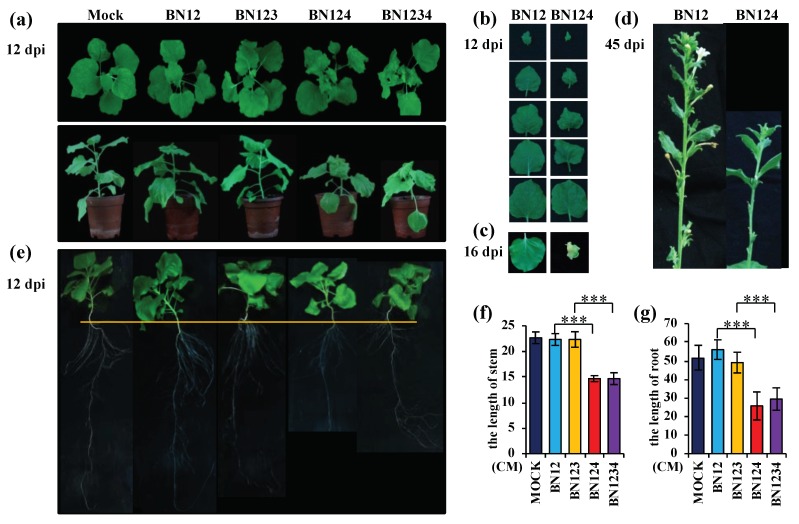
Stunting, leaf curling, and floral and root development defects in *Nicotiana benthamiana*, induced by *Beet necrotic yellow vein virus* (BNYVV), requires the presence of RNA4. BNYVV infections containing RNA4 induced; (**a**) stunting and leaf curling, (**b**) leaf area reduction, (**c**) leaf senescence, (**d**) floral development defects, and (**e**) root development defects. (**d**) We observed more than seven full capsules formed at the base of flowers with normal length in BN12-infected plants; in contrast, there were about four small, shriveled, deformed capsules at the base of abnormally short flowers. Statistical analysis of the stem and root length of different plants, shown in (**a**) and (**e**), are indicated in (**f**), and (**g**), respectively. Error bars represent standard deviation of three individual plants (*n* = 3); *** *p* < 0.01.

Analysis of different treatments including Mock-, BN12 (RNAs1+2)-, BN123 (RNAs1+2+3)-, BN124 (RNAs1+2+4)-, and BN1234 (RNAs1+2+3+4)-infected *N. benthamiana* revealed that BNYVV-induced symptoms were exacerbated in the presence of RNA4, which were characterized by stunting, downward leaf curling (Figure 1a), and leaf area reduction at 12 days post-inoculation (dpi) (Figure 1b). In contrast, BN12 and BN123 infection produced mild symptoms in *N. benthamiana* (Figure 1a,b). Leaf chlorosis and senescence appeared on BN124-infected systemic leaves compared with BN12-infected leaves at 16 dpi (Figure 1c). Development defects, including short stem and low seeding rate, occurred in BN124-infected plants at 45 dpi (Figure 1d,f). Investigations into root development using Hoagland’s nutrient solution revealed that the of root length of *N. benthamiana* inoculated with either BN124 or BN1234 were shorter than those of Mock-, BN12-, or BN123-inoculated plants at 12 dpi (Figure 1e,g). Together, these results indicated that BNYVV RNA4 plays a major role in induction of stunting, leaf curling, and floral and root development defects in *N. benthamiana* during BNYVV infection.

### 3.2. Microarray Analysis of miRNAome in BNYVV-Infected N. benthamiana

A microarray analysis of healthy (L) and BN1234-inoculated *N. benthamiana* (VL) was performed by LC Sciences (Hangzhou, China) using 1596 predicted miRNA probes. We obtained 388 miRNAs with a hybrid signal >500 in L or VL microarray library, including 285 conserved miRNAs belonging to 50 families and 103 novel miRNAs. Among them, miR169, miR393, miR394, miR395, and miR399 showed low expression level (hybrid signal below 500 in L and VL microarray library) in BNYVV-infected *N. benthamiana* [38]. However, due to their common existence in all terrestrial species [39,40], these miRNAs were also included for subsequent assays. 

#### 3.2.1. Phylogenetic Analysis of the microRNAome of *N. benthamiana*

**Figure 2:**
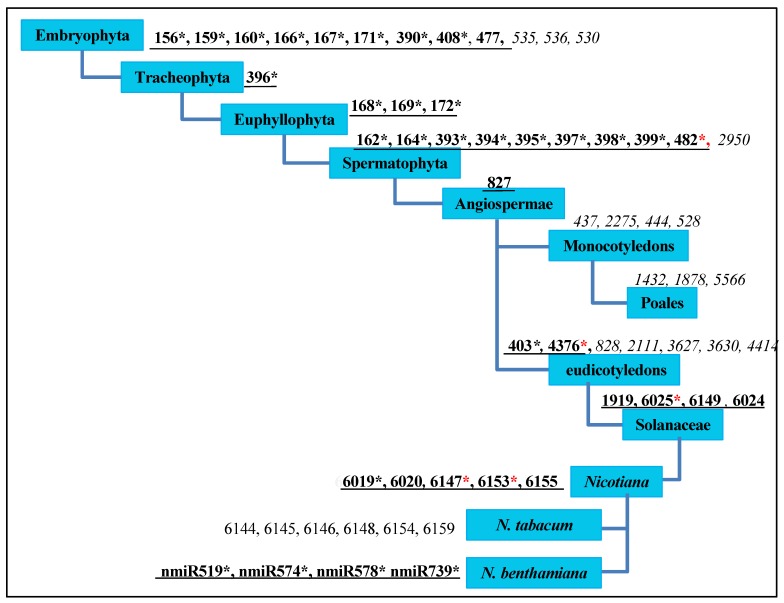
The distribution of microRNAs (miRNAs) of *N. benthamiana* from the phylogenetic perspective of plant miRNAs. The phylogenetic perspective was proposed by Taylor et al. [41]; the graphic mode was generated, according to the model constructed by Yin et al. [42]. Underlined black bold numbers indicate miRNA families in *N. benthamiana*; italics indicate missing miRNAs; * indicates that miRNAs belong to at least one member of an identified miRNA family; black * represents miRNAs that have been reported previously; red * represents newly identified miRNAs.

To gain an evolutionary insight into the *N. benthamiana* microRNAome from the microarray analysis, we investigated the miRNAs’ distribution within the phylogenetic perspective of plant miRNAs proposed by Taylor et al. [41]. Through sequence analyses, we identified 25 out of 34 conserved miRNA families in Eudicotyledons, four linage-specific miRNAs in *Solanaceae* (miR1919, miR6149, miR6024, and miR6025), and five in genus *Nicotiana* (miR6019, miR6020, miR6147, miR6153, and miR6155). Among all of these, miR482*, miR4376*, miR6025*, miR6147*, and miR6153* were identified for the first time and they were induced by BNYVV infection (Figure 2).

Nine miRNAs were missing in *N. benthamiana*: miR530, miR535, miR536, miR2950, miR828, miR2111, miR3627, miR3630, and miR4414 (Figure 2) [38,43,44], which are not identified or have a very low expression in *Nicotiana tabacum*, *Solanum lycopersicum* and *Solanum tuberosum*, similar to that of stu-miR530 and stu-miR3627 (reads < 10) [45], implying that the missing or low expression of these nine miRNAs might be a common characteristic within the *Solanaceae*.

In summary, phylogenetic analysis of the identified *N. benthamiana* miRNAs provided useful information for miRNA research in other species of the genus *Nicotiana,* as well as for plant evolutionary analyses.

#### 3.2.2. Species-Specific miRNAs in *N. benthamiana*

Eight species-specific miRNAs were identified in *N. benthamiana* (Table 1). Firstly, 103 potential novel miRNAs (hybrid signal > 500 in L or VL library) were re-evaluated according to the stringent criteria reported by Taylor et al. [41]. nmiR519-5p, nmiR574-5p, nmiR578-5p, and nmiR739-5p, and their corresponding miRNA-3p were found to meet the screening criteria with a minimum free energy index (MFEI) > 1 (Table 1). All these four miRNAs have been reported previously [43,44], and the secondary structures of the precursors are shown in Figures S1–S4. Analysis of their precursor sequences by searching the NCBI public database revealed that none of them were identical to other plant species data sets including *N. tabacum*, suggesting that these eight miRNAs are species-specific and independently evolved within *N. benthamiana*.

*N. benthamiana* and *N. tabacum* have their own species-specific miRNAs. *N. benthamiana* does not have miR6144, miR6145, miR6146, miR6148, miR6154, and miR6159, which were originally designated as lineage-specific miRNAs in genus *Nicotiana* [38,43,44,46,47,48,49] (Figure 2). They are species-specific miRNAs in *N. tabacum*. These results indicate that plants with a phylogenetically close relationship, such as *N. benthamiana* and *N. tabacum* [50], have their own distinct miRNAs.

### 3.3. Differential Expression Profile of miRNAs

The microarray analysis revealed that 129 miRNAs (signal > 500 and *p*-value < 0.1 in L or VL libraries) were differentially regulated by at least a 1.5-fold change (FC) in BNYVV-infected *N. benthamiana* leaves compared with the mock-inoculated control (|log_2_Ratio (VL/L)| > 0.6). Among these miRNAs, 94 were induced and 35 were inhibited (Table S1).

**Figure 3:**
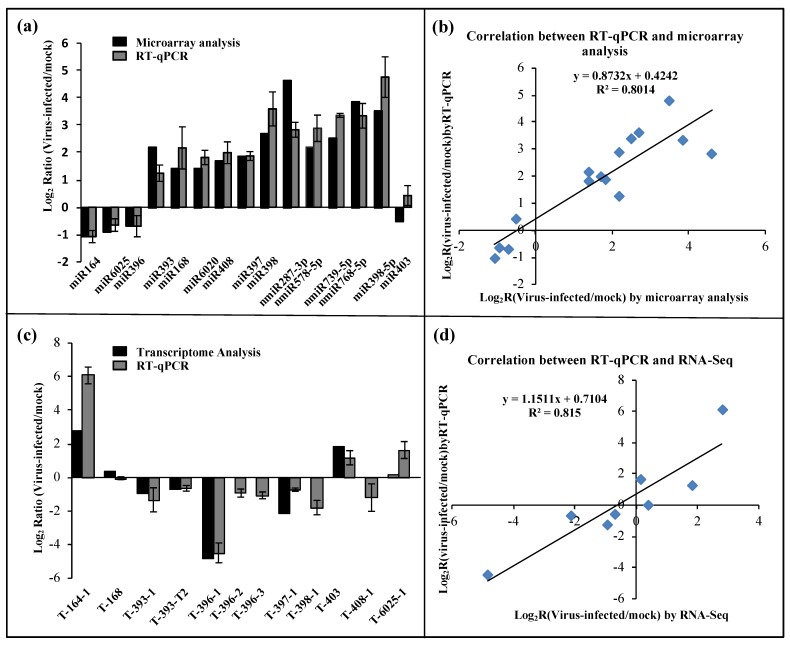
Differentially expressed miRNAs and their putative targets in mock- and BN1234-infected *N. benthamiana*. (**a**) Validation of the relative expression levels of selected miRNAs in response to BN1234, as determined by stem-loop quantitative real-time PCR (qRT-PCR) (gray), and microarray analysis (black). (**b**) Correlation of the expression ratio of selected miRNAs measured by stem-loop qRT-PCR and microarray analysis. (**c**) Validation of the relative expression levels of selected targets in response to BN1234, as determined by qRT-PCR (gray) and RNA-Seq (black) [10]. (**d**) Correlation of the expression ratio of selected targets measured by qRT-PCR and RNA-Seq [10]. *EF1A* was used as the internal reference control. The experiments were independently repeated three times; error bars represent the standard error of the mean (*n* = 3).

To evaluate the reliability of the results generated by the microarray analysis, stem-loop quantitative reverse-transcription Real-time PCR (qRT-PCR) was performed. We evaluated the expression patterns of 11 known miRNAs (miR164, miR168, miR393, miR396, miR397, miR398-3P/5P, miR403, miR408, miR6020, and miR6025) and four novel miRNAs (nmiR287-3p, nmiR578-5p, nmiR739-5p, and nmiR768-5p). A high correlation (*R*^2^ = 0.85) was found between the stem-loop qRT-PCR and microarray analysis (Figure 3a,b). Thus, the alterations in miRNA expression detected by the microarray analysis could reflect the actual miRNA expression changes between mock- and BN1234-infected plants. miRNAs were selectively regulated by BN1234 infection. For example, miR164, miR396, miR319, and miR393 were inhibited, and miR168, miR390, miR393, miR397, and miR398 were induced (Table S1).

During the processing of primary miRNA comprised of the miRNA-3p/miRNA-5p duplex, one of them enters into the RNA-induced silencing complex (RISC) to modulate target gene expression; the other one is degraded by an unclear mechanism, which is also called miRNA*. Among miRNAs/miRNAs* obtained from this microarray analysis, the amount of miRNA* was lower than corresponding miRNA in healthy *N. benthamiana* in contrast to the high accumulation of miRNAs* present in BN1234-infected *N. benthamiana*, regardless of the expression of their corresponding miRNAs changes (Figure S5). These results suggested that BN1234 infection may interfere with miRNA synthesis pathways through changing the expression of some miRNAs or impacting miRNA/miRNA* duplex stabilization.

### 3.4. Differential Expression of Target Genes of BN1234-Responsive miRNAs

To investigate the potential regulatory roles of the detected BN1234-responsive miRNAs, the miRNA targets were annotated by standard settings of psRNATarget [34] with a maximum expectation value 3.0 using the genome of *N. benthamiana* as a reference, and 645 targets were obtained (Table S2). Our results consistently revealed that plant-conserved miRNAs regulate similar targets across various species [51], such as miR156 targets the *Squamosal promoter-binding-like* gene, and miR162′s endoribonuclease Dicer homolog 1 and miR393′s transport inhibitor response 1. The expression pattern of 11 targets of eight miRNAs was examined by qRT-PCR, followed by comparison with the results of transcriptome sequencing about mock-Vs BN1234-inoculated plants, as reported previously [10]. The results showed a high correlation (*R*^2^ = 0.8313) between qRT-PCR and transcriptome sequencing (Figure 3c,d). These results suggested that the alterations in target expression are correlated with the changes in miRNAs during BNYVV infection, and the differential expression of these miRNAs are consistent with the results of transcriptome sequencing as performed previously [10]. 

**Figure 4:**
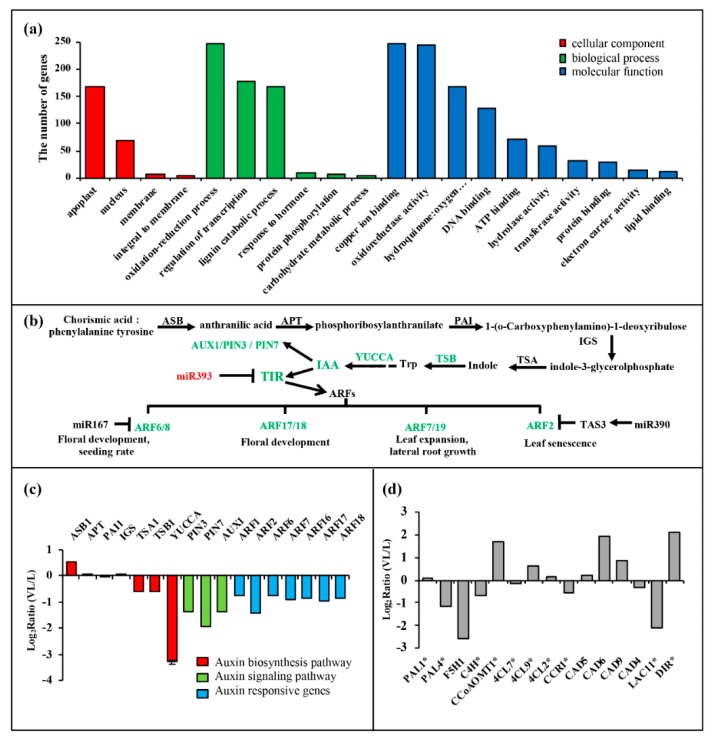
Gene ontology (GO) analysis of the predicted miRNA target genes and expression pattern of two signaling pathways that may contribute to the symptom induction by BNYVV. (**a**) GO analysis of predicted targets of differentially expressed miRNAs; (**b**) the expression changes of auxin signaling pathway caused by BNYVV infection; (**c**) the expression pattern of genes belonging to the auxin signaling pathway from transcriptome analysis, and IAA synthetic gene *YUCCA* from qRT-PCR analysis of mock- and BN1234-infected *N. benthamiana*. (**d**) The expression pattern of genes that function in lignin biosynthesis pathway. * represents the differential expression of this gene could significantly change the content of lignin [54,55,56,57,58,59,60].

In BN1234-infected *N. benthamiana*, miR393, miR397, miR398, and miR408 were induced, and their targets were inhibited correspondingly. Similarly, miR164 and miR6025 were inhibited, and their targets were induced correspondingly. However, we noted that miR396 and their targets were all down-regulated; miR168 was up-regulated, and its target expression level did not change significantly, which might be due to the selective regulation of some miRNAs and their targets by BNYVV infection. Gene ontology (GO) analysis was performed, and these target genes involve 78 items, including “response to hormone” and “lignin catabolic process” (Figure 4a). The hormone signaling pathway plays an important regulatory role in the growth and development of plants. Studies showed that plant virus-induced symptoms are related to hormone dysfunction. For example, the stunting symptom in RSV-infected rice was induced by inhibiting synthesis of GA and IAA [24,25]. We then analyzed the target genes involved in the hormone signaling pathway. The results showed miR160 was up-regulated and its target genes *ARF17/18* were inhibited in BNYVV-infected *N. benthamiana* leaves (Figure 4b,c). Previous studies have shown that the differential expression of miR167 and its target genes is related to the development of floral organs [52]. The alterations in miR167 and its target genes, during BNYVV infection, as mentioned above, suggested their potential roles in mediating the developmental defects in floral organs during BNYVV infection (Figure 1). A similar case was also observed for miR393, which targets the transport inhibitor response protein (TIR) and other auxin response factors (ARFs) [53]. The upregulation of miR393 during BNYVV infection may also contribute to the stunting and the development defects in root and floral organ (Figure 1).

ASB, Anthranilic acid synthase; APT, anthranilate phosphoribosyl transferase; PAI, phosphoribosyl-lanthranilate isomerase 1; IGS, indole-3-glycerolphosphatesynthase; TSA, tryptophan synthase α chain; TSB, tryptophan synthase β-subunit 1; YUCCA, flavin monooxygenase-like enzyme (FMO); the YUCCA gene is a rate-limiting enzyme in the auxin biosynthesis pathway. PAL, phenylalanine-ammonialyase; F5H, Ferulate-5-hydroxylase; C4H, Cinnamate-4-hydroxylase; CCoAOMT, Caffeoyl-CoA O-methyltransferase; 4CL, 4-hydroxycinnamoy-CoA ligase; CCR, Cinnamoyl-CoA reductase; CAD, Cinnamyl-alcohol dehydrogenase; DIR, dirigent-like protein.

**Figure 5:**
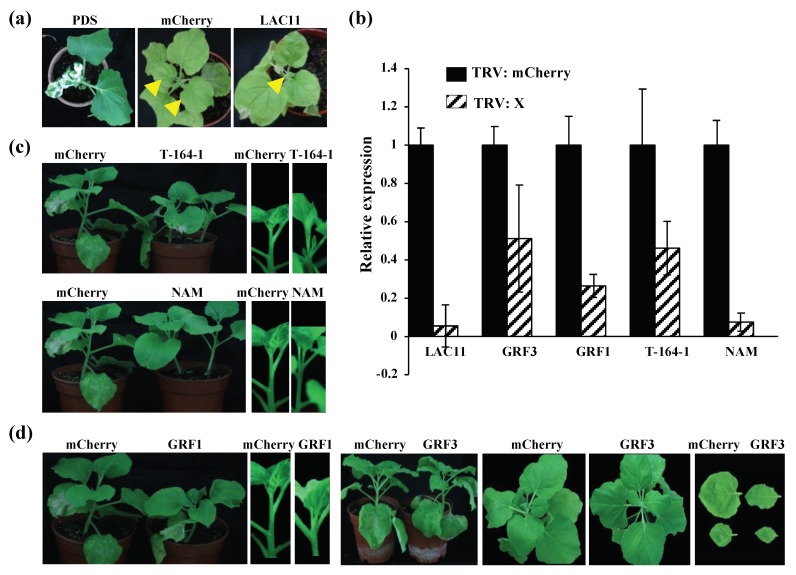
The phenotypes of silencing of the *LAC11*, *GRF1*, and *GRF3* genes at 14 days post-inoculation (dpi). (**a**) Silencing of *LAC11* inhibited the formation of new upper leaves; *N. benthamiana* plants agroinfiltrated with TRV:mCherry showed no obvious abnormality at 14 dpi. In contrast, silencing of LAC11 led to the pronounced inhibition of plant apical meristem growth (see the leaves indicated by the yellow arrowheads). (**b**) qRT-PCR analysis confirmed the silencing of these target genes as described in (**a**,**c**,**d**). (**c**) Silencing of *T-164-1* interfered with the growth of apical meristems, and there were no obvious phenotypes in TRV:NAM-inoculated plants. *NAM* and *T-164-1* are two different genes of NAC (NAM/ATAF/CUC) gene family. (**c**) Silencing of *GRF1* caused the dwarf symptom and inhibited the growth of leaves; TRV:mCherry- and TRV:PDS (phytoene desaturase gene)-inoculated *N. benthamiana* served as the negative, and positive controls, respectively.

In addition, miR397 was induced, and its target gene, *LAC11*, was significantly reduced in BNYVV-infected *N. benthamiana* (Figure 3). LAC11 encodes the laccase that catalyzes the oxidation of lignin monomers into polymers at cell wall, and participates in the formation of cell walls [61]. To investigate the function of the down-regulation of *LAC11* in the symptom induction during BNYVV infection, *Tobacco rattle virus* -induced gene silencing (VIGS) was used for knocking down *LAC11*, and the phenotype of VIGS-treated plants was monitored. The results showed that silencing of LAC11 considerably inhibited plant apical meristem growth in comparison to the TRV:mCherry-inoculated control plants, because silencing of *LAC11* inhibited the growth of new upper leaves (see the leaves indicated by the yellow arrowheads inset Figure 5a). qRT-PCR confirmed that the expression of LAC11 was significantly down-regulated at 14 days post infiltration (Figure 5b). Therefore, these results suggested that an association exists in the up-regulation of miR397 and subsequent down-regulation of its target genes with the appearance of stunting symptoms in BN1234-infected *N. benthamiana*. 

### 3.5. miRNAs-Targeted Genes Involved in Hormone Signaling Function in the Symptom Induction of BNYVV

BNYVV caused leaf curling and stunting in *N. benthamiana*. As plant hormones play an important regulatory role in plant growth and development, we analyzed the effect of the miRNAs involved in the hormone signaling pathway on the formation of symptoms during BNYVV infection. 

#### 3.5.1. BN1234 Infection Interferes with miR164-NACs-ETH Pathway

**Figure 6:**
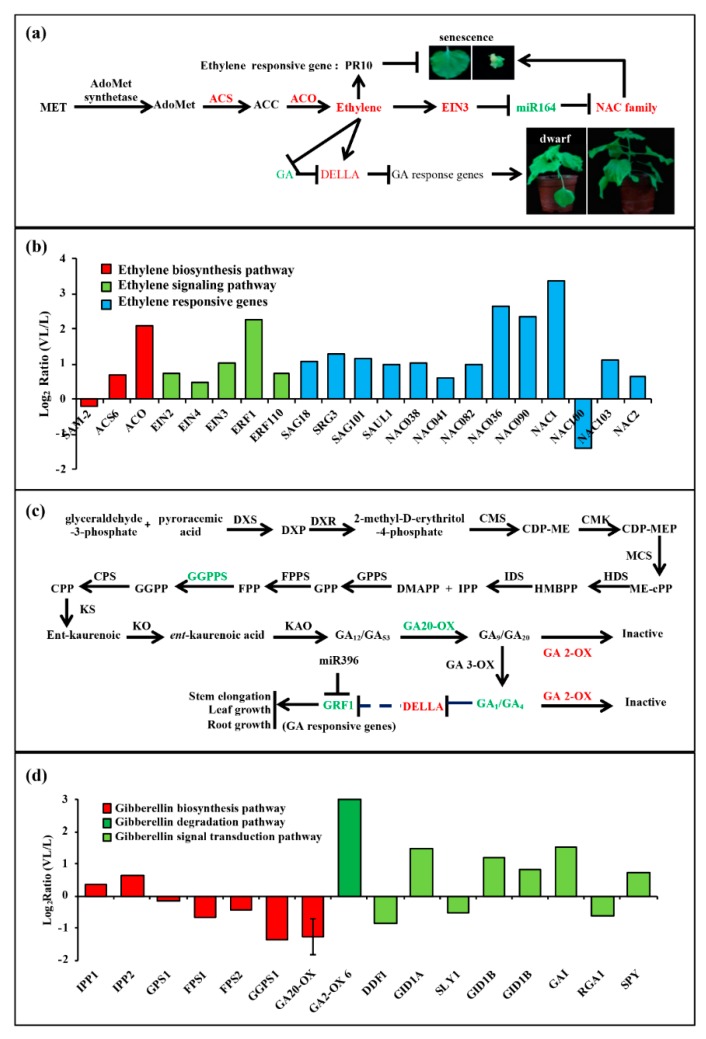
The changes in ethylene and gibberellin signal pathways caused by BN1234 infection. The expression pattern of genes involved in (**a**,**b**) ethylene signal pathway and (**c**,**d**) gibberellin signal pathway from transcriptome analysis, except GA20-OX, which was identified from qRT-PCR analysis of mock- and BN1234-inoculated *N. benthamiana*.

BNYVV infection induces the ethylene signaling pathway. The ethylene biosynthetic pathway is methionine (Met)-S-methionine (SAM)-1-amino cyclopropane-1-carboxylic acid (ACC)-ethylene (Figure 6a), which involves S-adenosylmethionine synthetase (SAMS), ACC synthetase (ACS), and ACC oxidase (ACO) [62]. The transcriptome sequencing results showed that the expression of SAMS was almost unchanged, ACS and ACO both increased, which is consistent with the upregulation of EIN2, EIN3, and ERFs in BNYVV-infected *N. benthamiana* [63]. In addition, PR-10, the ethylene response factor (Figure S6), was up-regulated to alleviate the senescence symptoms caused by increased-content ethylene, which also supported that BNYVV RNA4 infection induced ethylene biosynthesis (Figure 6b) [9,64].

The miR164 down-regulated by BNYVV infection may positively regulate apical meristem growth. miR164 expression decreased and its target gene, *T-164-1* (NAC21/22), was significantly induced in BNYVV-infected *N. benthamiana*. TRV-induced gene silencing (VIGS) was used to investigate the function of T-164-1, and TRV:mCherry- or TRV:NAM-inoculated plants were used as the negative control. NAM and T-164-1 (NAC21/22) are proteins of NAC family. The results showed that the growth of plant apical meristem appeared normal in TRV:mCherry-inoculated and TRV:NAM-inoculated plants. In contrast, silencing of T-164-1 (NAC21/22) significantly inhibited the growth of plant apical meristem, and enhanced lateral bud growth (Figure 5c). qRT-PCR confirmed that the expression of T-164-1 and NAM were significantly down-regulated at 14 dpi (Figure 5b). Thus, these results suggested that BN1234 represses the expression of miR164, which may compromise the inhibition effect caused by some other genes, such as *miR397-LACs*, thus maintaining the apical meristem growth to some extent.

#### 3.5.2. BN1234 Infection Changed miR396-GRFs-GA Pathway

BN1234 infection inhibited the gibberellin (GA) synthesis and signal transduction pathway. GA is a kind of terpenoid, and geranyl pyrophosphate and isopentenyl pyrophosphate are the most important precursor compounds for gibberellin biosynthesis (Figure 6c) [65]. Isopentenyl pyrophosphate: dimethylallyl pyrophosphate isomerase (IDS) and geranylgeranyl pyrophosphate synthase (GGPPS) were inhibited by RNA4 from BNYVV. GA20-oxidase, which catalyzes the conversion of inactive GA12 into active form, decreased, and GA2-oxidase, which catalyzes the conversion of active GA9/GA20 and GA4/GA1 into inactive GA51/GA29 and GA34/GA8, increased [66]. Therefore, the biosynthesis of the active form of GA was inhibited, but the degradation was enhanced in BNYVV-infected *N. benthamiana*. DELLA protein, a suppressor in GA signal transduction pathway, was induced (Figure 6d), which is consistent with results described previously [10]. In summary, these results indicated that the GA synthesis and signal transduction pathway is negatively regulated by BNYVV infection.

*GRF1* is the responsive gene of the GA pathway [67]. The GA signal pathway was inhibited, and the expression of GRF1 was down-regulated in BN1234-infected *N. benthamiana* (Figure 3c). Compared with TRV:mCherry- and TRV:GRF3-treated plants, silencing GRF1 significantly inhibited the elongation of the stem and caused the dwarf symptom (Figure 5d,b). Altogether, these results suggested that the down-regulation of GA-GRF1 pathway is strongly correlated with the formation of dwarf symptoms during BNYVV infection.

### 3.6. miR168 and miR398 Induction Commonly Occurred During Plant–Virus Interaction 

One of the main attributes of plants is the activation of a defense response against virus infection, including oxygen burst, induced synthesis of pathogenesis-related proteins, and so on; most plant viruses have viral suppressors of RNA silencing (VSRs) to counteract the host RNA silencing defense response. To investigate whether some commonalities exist in the microRNAome of plants during the interplay between plants and viruses, a comparison of our data with 28 other previously reported plant*–*virus combinations indicated that miR168 and miR398 are up-regulated in a variety of plants during infection with different viruses (Table 2). 

#### 3.6.1. Induction of miR168 during Virus Infection May be Related to VSRs

miR168 was induced in *N. benthamiana* infected by BNYVV. Up-regulated miR168 was found in 22 other combinations of different plants and VSRs (Table 1). Two exceptions were found in *N. benthamiana*: BBSV and CymRSVP19stop interactions. BBSV was not reported to encode a VSR, and a premature stop codon was introduced into the CymRSV genome to inactivate the expression of p19 VSR (CymRSVP19stop) [31,68]. 

The up-regulation of miR168 is not related to the host species and the mode in which different VSRs act. For example, all the combinations of BNYVV–*N. benthamiana*/–*Beta macrocarpa*, TCV–*N. benthamiana*/–*Arabidopsis thaliana*, and CMV–*N. tabacum*/–*Solanum lycopersicum* showed induction of miR168, but these viruses encode different VSRs. P38 encoded by TCV disturbs DCL by binding long dsRNAs (double-stranded RNAs); CMV-encoded 2b preferentially binds to siRNAs (Small interfering RNAs) to interfere their loading into AGO1. βC1, the VSR of TYLCCNV, induces the expression of endogenous suppressors of RNA silencing, *Nicotiana benthamiana* calmodulin-like protein (Nbrgs-CaM), to fight against the host RNA-silencing-mediated defense [78,79,80]. 

Altogether, the upregulation of miR168 commonly occurs during plant–virus interactions, which may be associated with the virus-encoded VSRs.

#### 3.6.2. miR398 Induction During Plant–Virus Induction Could Trigger Plant Defense Response

Analysis of the differential expression of miRNAs in 12 combinations of plants and viruses showed that miR398 was induced in all combinations (Table 1), irrespective of virus or plant species. For example, the expression of miR398 is up-regulated in *N. benthamiana* infected by seven viruses and in two different plants infected by TMV.

**Figure 7:**
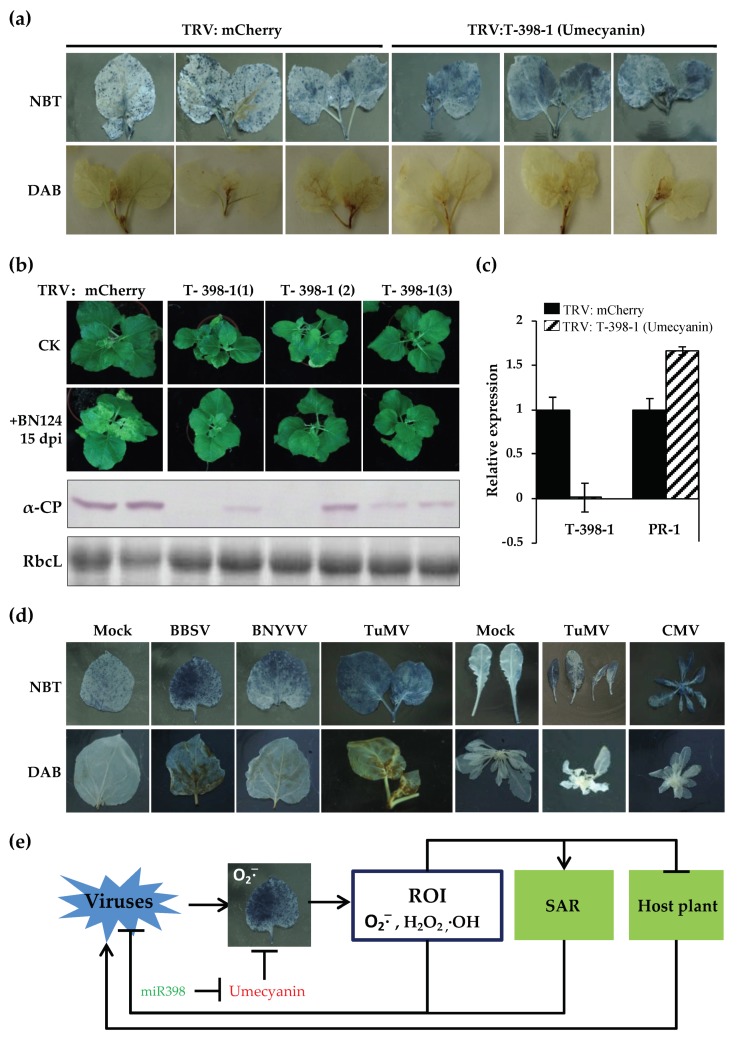
Silencing of miR398′s target gene *umecyanin* enhances plant resistance against viruses, which is related to the increased level of O_2_^−^. (**a**) The level of O_2_^−^ was higher in TRV: umecyanin-treated *N. benthamiana* than that of TRV:mCherry-treated plants. (**b**) Silencing of miR398′s target gene *umecyanin* could enhance plant resistance against BNYVV; (**c**) qRT-PCR analysis was used to confirm the silencing of *umecyanin* and the upregulation of *PR-1* in TRV:umecyanin-inoculated *N. benthamiana* plants. (**d**) The level of O_2_^−^ increased in different viruses-infected plants. (**e**) A proposed model for the miR398- and O_2_^−^-mediated plant defense against virus infection.

Umecyanin is one of targets of miR398 (T-398) in *N. benthamiana* [38,81] and is involved in redox reactions occurring during primary defense responses in plants and/or in lignin formation. miR398 expression is up-regulated in *N. benthamiana* infected by BN1234, and the expression of the target gene *umecyanin* was decreased (Figure 3a,c). To investigate the role of umecyanin in the process of BNYVV infection, TRV-induced gene silencing (VIGS) was used to downregulate the expression of umecyanin. The expression of umecyanin was investigated by qRT-PCR and the concentration of O_2_^−^ and virus were detected at 6 dpi. NBT (nitroblue tetrazolium) or DAB (diaminobenzidine) staining was performed to analyze the level of O_2_^−^ or H_2_O_2_. The results showed that the concentration of O_2_^−^ was higher in plants when umecyanin was silenced (Figure 7a). Both umecyanin-silenced and vector control plants were challenged with BN124 at 7 dpi. Western blot analysis showed that the accumulation of BNYVV coat protein was lower in the systemic leaves of *N. benthamiana* with silencing of umecyanin at 25 dpi in comparison to that bombarded with the empty vector control (Figure 7b), indicating that silencing of miR398′s target gene *umecyanin* could enhance plant resistance against viruses. qRT-PCR analysis confirmed that the umecyanin mRNA was significantly reduced in TRV:umecyanin-inoculated plants, compared with TRV:mCherry-inoculated control plants (Figure 7c). These results suggested that the induction of miR398 during BNYVV infection is favorable for the activation of plant defense responses.

## 4. Discussion

We performed microarray analysis using 1596 probes to investigate microRNAs of *N. bethamiana* and their responses to BNYVV infection. A total of 388 miRNAs were identified with hybrid signal >500 in L or VL microarray library, and the phylogenetic analysis of the microRNAome of *N. benthamiana* was performed in combination with data from previous reports. The results showed that there were 25 out of 34 miRNA families in Eudicotyledons, and the loss or low abundance of nine miRNAs (miR530, miR535, miR536, miR2950, miR828, miR2111, miR3627, miR3630, and miR4414) might be a common feature of plants in the *Solanaceae*. In addition, *N. tabacum* and *N. benthamiana* have their own species-specific miRNAs. Eight species-specific miRNAs were obtained in *N. benthamiana*. We identified 129 differentially expressed miRNAs by microarray analysis, and their targets were predicted accordingly and analyzed.

Leaf curling, induction of stunting, and root and floral development defects are dependent on the presence of BNYVV RNA4. Hormones play an important role in the growth and development of plants. Therefore, a systematic correlation analysis was performed on the changes in the expression of genes and miRNAs involved in hormone signaling pathways. Among them, IAA synthesis was inhibited. miR160 and miR393, which participate in the IAA signaling pathway, were correspondingly induced and ARFs were decreased accordingly. These factors may be associated with the defects in the root and flower organs. The ethylene synthesis pathway was activated. Down-regulation of miR164 and up-regulation of NAC21/22 involved in the ethylene signal transduction pathway was observed in BNYVV-infected leaves. miR164-NAC21/22, together with miR397-LACs, could regulate apical meristem growth, and both factors might be associated with the formation of stunting phenotypes. GA synthesis was inhibited, the expression of GA response factor GRF1 (one target of miR396) also decreased, which may play a role in the formation of stunting symptoms. GRF1 is the target of miR396. The expression of both GRF1 and miR396 in the plants infected with BNYVV was reduced (Figure 3a,c), and the differential expression of these target genes did not appear to be negatively related with their respective miRNAs. Previous studies also showed that no simple linear gene expression regulatory mechanisms exist, but rather multi-faceted regulatory networks of varying complexity in the miRNA396-GRF regulatory unit, and the GRF gene family is subject to a regulatory mechanism that are independent of miR396 [82]. Therefore, the down-regulation of miR396 does not necessarily result in the increase of GRF1 expression.

Our findings revealed that BNYVV infection induced ethylene biosynthesis but repressed GA signal pathway in the infected *N. benthamiana* leaves, consistent with the results from transcriptome analysis of BNYVV-infected *N. benthamiana* leaves reported previously [10]. However, Fernando Gil et al. reported that the ethylene signal transduction pathway is strongly suppressed in the BNYVV-infected root tissues of sugar beet [11], and neither *B. macrocarpa* nor *B. vulgaris* was reported to show changes in the genes of the GA signal pathway during BNYVV-infection [7,11,12]. Instead, the auxin response is predominantly regulated in both *B. macrocarpa* and *B. vulgaris* during BNYVV infection [7,11,12], suggesting plant-species-specific hormonal responses to BNYVV. Notably, auxin increased considerably in sugar beet roots after BNYVV infection [11,12], whereas in the leaves of *B. macrocarpa*, the auxin response seems to be repressed, as evidenced by the upregulations of miR393 and consequent down-regulation of transport inhibitor response protein 1 [7]. Similarly, jasmonic acid content was proposed to be increased in the BNYVV-infected leaves of *Beta macrocarpa*, due to the down-regulation of miR319 that is involved in the JA biosynthesis pathway [7,22,83], but showed no obvious alteration in the roots of both *B. macrocarpa* and *B. vulgaris* during BNYVV infection [11]. These results implied that hormone signaling is regulated in a tissue-dependent manner during BNYVV infection. Regardless of plant species or plant tissues, the activation of the plant defense signaling including oxidative stress (e.g., Reactive oxygen species (ROS) response) and the up-regulation of pathogenesis-related genes (e.g., *PR-1*) is frequently observed during BNYVV infection [7,11,12], indicating a shared response to BNYVV infection aong phylogenetically-distant plant species. More studies need to be performed in the future to further illustrate how these distinct signaling pathways are coordinately regulated during BNYVV infection.

The up-regulation of miR168 and miR398 may commonly occur in virus-infected plants. The increased expression of miR168 may be related to VSRs, which are often primary pathogenicity determinants of viruses. *AGO1* is the target gene of miR168. The expression of miR168 was significantly up-regulated in BNYVV-infected *N. benthamiana*. However, the differential expression of AGO1 mRNA was not detected. Previous studies also reported that changes in the expression of miR168 and AGO1 mRNA are not always negatively correlated [84,85], Nevertheless, the decrease in the expression of AGO1 protein could alleviate the host RNA-silencing-mediated defense to benefit for the infection of the virus [68]. 

The up-regulated expression of miR398 may be associated with the primary defense response among different plant species. Its target genes include *umecyanin* (in *N. benthamiana*) or Cu/Zn-superoxide dismutases (*CSDs*) and a copper chaperone for both CSD1 and CSD2 (*CCS*) in *Arabidopsis*, which are all related to the redox process in the activation of primary defense response of plants. The increased expression of miR398 and the corresponding decreased expression of its target genes is favorable for the activation of plant defense responses against virus infection, which could be reflected, at least to some extent, by the enhanced systemic acquired resistance after knocking down the *umecyanin* expression.

O_2_^−^, H_2_O_2_, and ·OH radicals are three main forms of reactive oxygen intermediates (ROI), and play an important role in plant defense responses [86,87,88,89,90]. In plants, O_2_^−^ can be converted to H_2_O_2_ spontaneously or in the presence of superoxide dismutase (SOD). O_2_^−^ reacts with H_2_O_2_ to produce ·OH radicals via Fenton’s reactions. The accumulation of O_2_^−^ can promote the formation of ·OH radicals. The accumulation of O_2_^−^ causes the increase in other reactive oxygen intermediates. High concentrations of reactive oxygen in plants might directly damage the nucleic acids of the virus to inhibit the replication of the virus; conversely, it can activate the plant disease resistance system. For example, *PR-1* mRNA was induced in TRV:umecyanin-treated plants (Figure 7c).

The concentration of O_2_^−^ increased in TRV:umecyanin-treated *N. benthamiana* and *csd1*, *csd2* and *ccs* (target genes of miR398 of *Arabidopsis*) mutant *Arabidopsis* [37] (Figure 7a), consistent with the report of Guan et al. [37]. O_2_^−^ was also induced in *Beet black scorch virus*-, *Cucumber mosaic virus*-, or *Turnip mosaic virus*-infected *N. benthamiana* or *Arabidopsis thaliana* (Figure 7d). In summary, we proposed a model where O_2_^−^ is activated during virus infection, and O_2_^−^ scavenging activity of plants is inhibited due to increased expression of miR398. All the above factors directly or indirectly enhance the antiviral response of plants (Figure 7e). 

In this work, a regulatory module consisting of miRNAs, their corresponding target genes, and the related signaling pathways was established, which strongly supports the functional involvement of miRNAs in mediating the symptom production by BNYVV. Our results extend the current knowledge of the mechanisms underlying BNYVV pathogenesis and are useful for the development of new antiviral strategies to control BNYVV-induced plant diseases.

## Figures and Tables

**Table 1:** Summary of species-specific miRNAs identified in *N. benthamiana*.

miRNA Name	Length	MFEI	Norm-L	Norm-VL	*Nicotiana benthamiana*	*Nicotiana tabacum*	Other Species	Other Paper
nmiR519-3p	21	1.3	268	262	Found	Not found	Not found	Y
nmiR519-5p	21	1.3	1900	1471	Found	Not found	Not found	Y
nmiR578-3p	21	1.4	0	312	Found	Not found	Not found	Y
nmiR578-5p	21	1.4	112	825	Found	Not found	Not found	Y
nmiR574-3p	21	1.2	94	93	Found	Not found	Not found	Y
nmiR574-5p	21	1.2	654	275	Found	Not found	Not found	Y
nmiR739-3p	22	1.3	819	5216	Found	Not found	Not found	Y
nmiR739-5p	21	1.3	1058	10851	Found	Not found	Not found	Y

Notes: MFEI: minimal free energy index; Norm-L: reads of miRNAs in L library used health *N. benthamiana* leaves as samples after normalization of the signals using the locally weighted regression (LOWESS) filter; Norm-VL: reads of miRNAs in VL library used BN1234-infected *N. benthamiana* leaves as samples after normalization of the signals using the locally weighted regression (LOWESS) filter.

**Table 2:** The expression of miR168 and miR398 in different plants during virus infections.

Plant	Virus	Viral Suppressor of RNA Silencing	miR168	miR398	Reference
*Nicotiana benthamiana*	BBSV	-	↓	-	[31]
	Cym19Stop	-	↓	-	[68]
	CymRSV	p19	↑	-	[68]
	BNYVV	p14+p31	↑	↑	Figure 3a
	ACMV	AC4	↑	↑	[69]
	CLCuMV+B	AC4	↑	-	[69]
	CbLCuV	AC4	↑	↑	[69]
	TYLCV	V2	↑	-	[69]
	TYLCCNV+B	βC1	↑	↑	[43]
	TMV	Replicase large subunit	↑	↑	[44]
	TCV	p38	↑	-	[68]
	PVX	p25	↑	↑	[49,70]
	TEV	Hc-Pro	↑	-	[68]
	PVY	Hc-Pro	↑	↑	[71]
*Nicotiana tabacum*	PVX	P25	↑	-	[49]
	CMV	2b	↑	-	[49]
*Arabidopsis thaliana*	TMV	Replicase large subunit	↑	↑	[68,72]
	TCV	p38	↑	-	[73]
	CMV	2b	↑	-	[68]
	RMV	Replicase large subunit	↑	-	[68]
*Brassica rapa*	TuMV	HC-pro	↑	↑	[74]
*Oryza sativa*	RBSDV	unknown	-	↑	[75]
*Solanum lycopersicum*	PVX	p25	↑	-	[68]
	TMV	Replicase large subunit	↑	-	[68]
*Medicago truncatula*	SHMV	Replicase large subunit	↑	-	[68]
*Beta macrocarpa*	BNYVV	p14+p31	↑	-	[7]
*Chaenomeles sinensis*	PMeV	unknown	-	↑	[76]
*Vitis vinifera*	GLRaV-3	unknown	-	↑	[77]

Notes: BBSV: *Beet black scorch virus*; CymRSV: *Cymbidium mosaic virus*; BNYVV: *Beet necrotic yellow vein virus*; ACMV: *African cassava mosaec virus*; CLCuMV: *Cotton leaf curl Multan virus*; CbLCuV: *Cabbage leaf curl virus*; TYLCV: *Tomato yellow leaf curl virus*; TYLCCNV: *Tomato yellow leaf curl China virus*; TMV: *Tobacco mosaic virus*; TCV: *Turnip crinkle virus*; PVX: *Potato virus X*; TEV: *Tobacco etch virus*; PVY: *Potato virus Y*; CMV: *Cucumber mosaic virus*; RMV: *Ribbgrass mosaic virus*; TuMV: *Turnip mosaic virus*; RBSDV: *Rice black-streaked dwarf virus*; SHMV: *Sun-hemp mosaic virus*; PMeV: *Papaya meleira virus*; GLRaV-3: *Grapevine leafroll-associated virus 3*; Cym19Stop is the CymRSV mutant virus which is unable to express RNA silencing suppressor p19 CymRSV encoded; B: betasatellite; “↑” means up-regulated expression; “↓” means down-regulated expression; “-” means that there is no silencing suppressor in the virus; “/” means that there is no report in relevant literature.

## Supplementary Materials

Supplementary materials can be found at https://www.mdpi.com/1999-4915/12/3/310/s1.
