# Supplementary material for: Genome-Wide microRNA Profiling Using Oligonucleotide Microarray Reveals Regulatory Networks of microRNAs in Nicotiana benthamiana During Beet Necrotic Yellow Vein Virus Infection
Source: Viruses. 2020 Mar 12;12(3):310. doi: 10.3390/v12030310 (PMC7150760; doi:10.3390/v12030310)
Supplement: Supplementary file 1 [file viruses-12-00310-s001.zip › Figure S5-S8.pdf]

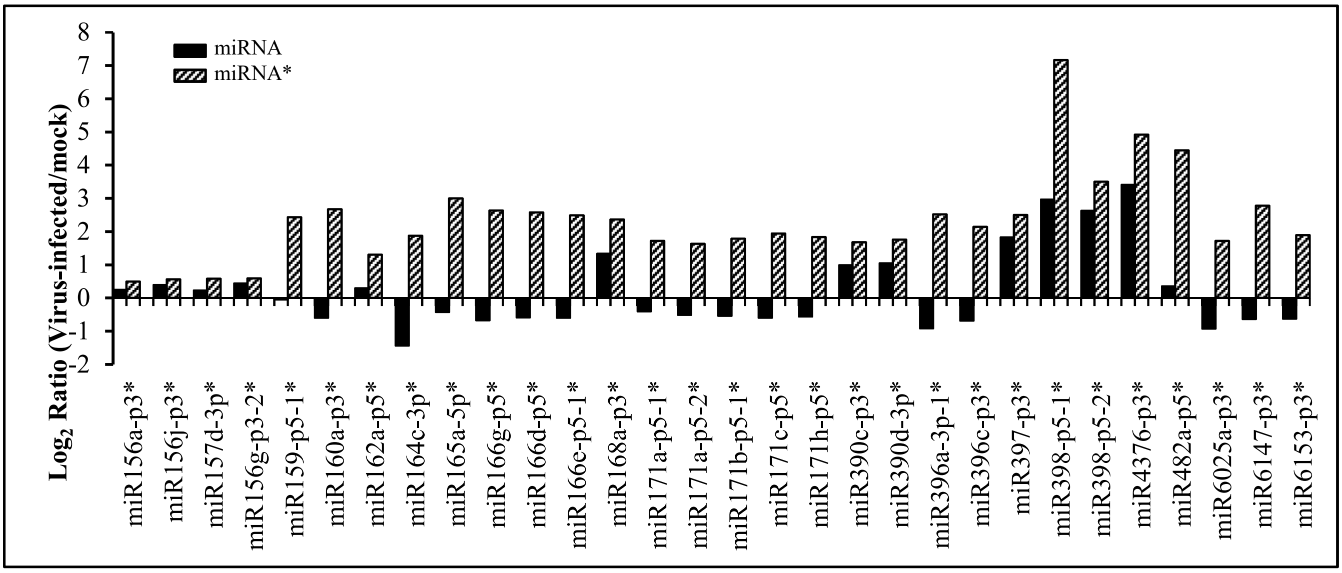

Figure S5. The expression pattern of some selected miRNA in BN1234-infected *N. benthamiana*.

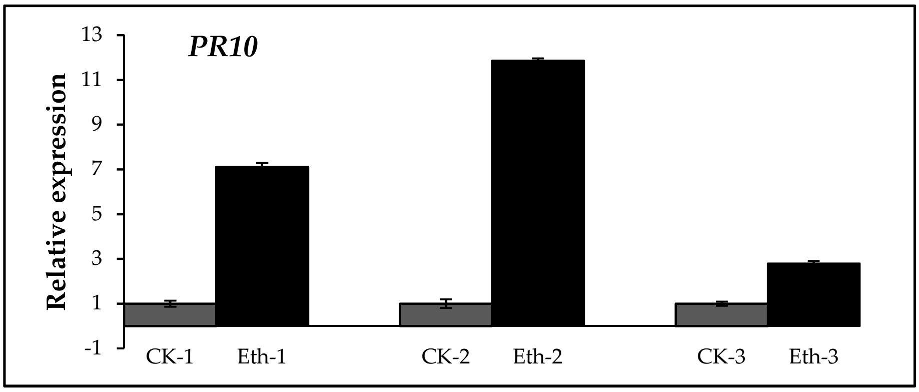

Figure S6. RT-qPCR analysis of *PR10* expression in *N. benthamiana* after ethylene treatment.

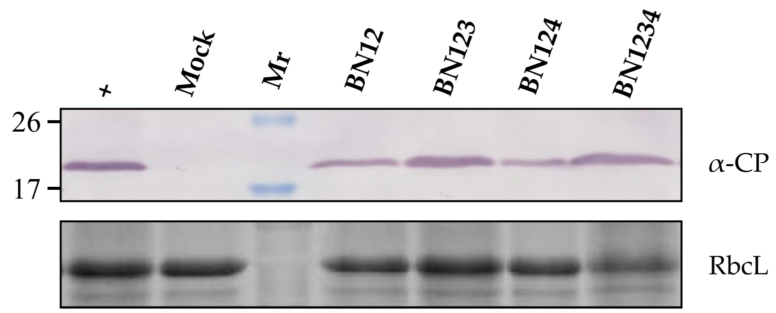

Figure S7. Western blot analysis of total protein extracts from the systemic leaves of *N. benthamiana* inoculated with various combinations of *in vitro* transcribed BNYVV RNAs. Antibody used for detection was indicated on the right and sizes (in kDa) of molecular weight markers are shown on the left. RbcL served as the loading control. "+" indicates purified BNYVV virions, which was served as the positive control.

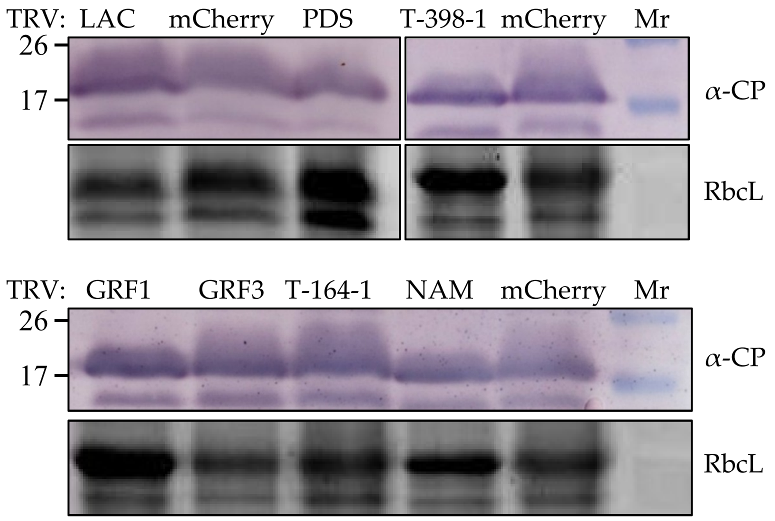

Figure S8. Western blot analysis of total protein extracts from the systemic leaves of *N. benthamiana* agroinfiltrated with various TRV-based silencing constructs. Antibody used for detection was indicated on the right and sizes (in kDa) of molecular weight markers are shown on the left. RbcL served as the loading control.
