# Supplementary figures and images for: Genome-Wide microRNA Profiling Using Oligonucleotide Microarray Reveals Regulatory Networks of microRNAs in Nicotiana benthamiana During Beet Necrotic Yellow Vein Virus Infection
Source: Viruses. 2020 Mar 12;12(3):310. doi: 10.3390/v12030310 (PMC7150760; doi:10.3390/v12030310)

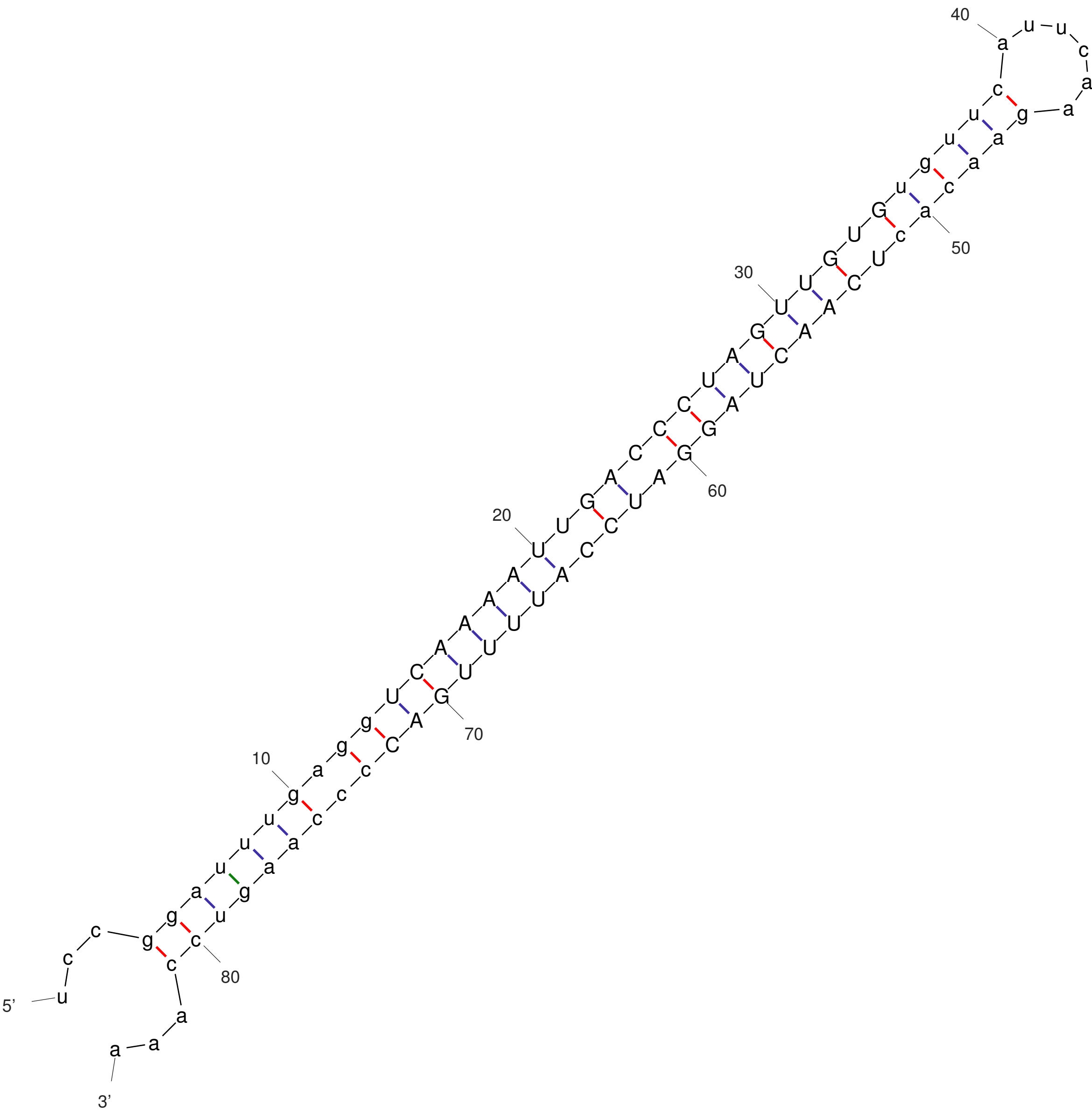

Supplement: Supplementary file 1 [file viruses-12-00310-s001.zip › Figure S1 miR519-5p and -3p.pdf]

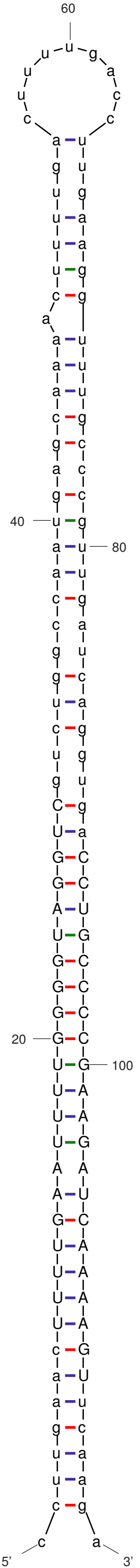

*dG = -66.90 [Initially -66.90] 18Mar08-09-38-30*

Supplement: Supplementary file 1 [file viruses-12-00310-s001.zip › Figure S2 miR574-5p and -3p.pdf]

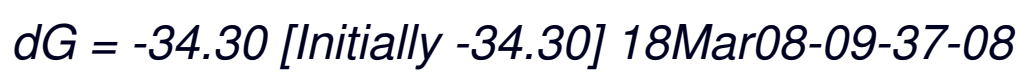

Supplement: Supplementary file 1 [file viruses-12-00310-s001.zip › Figure S3 miR578-5p and -3p.pdf]

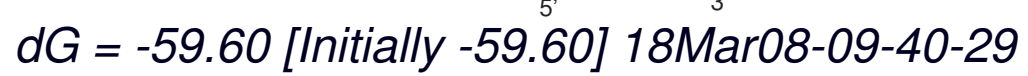

Supplement: Supplementary file 1 [file viruses-12-00310-s001.zip › Figure S4 miR739-5p and -3p.pdf]
